# Contrast-Enhanced Low-Mechanical-Index Endoscopic Ultrasound for the Evaluation of Focal Liver Lesions

**DOI:** 10.3390/diagnostics16162530

**Published:** 2026-08-11

**Authors:** Christoph F. Dietrich, Kathleen Möller, Bertrand Napoléon, Michael Hocke, Barbara Braden, Christian Jenssen

**Affiliations:** 1 Center of Education and Excellence, 3626 Thun, Switzerland; 2 Medical Department I—Gastroenterology, SANA Hospital Lichtenberg, 10365 Berlin, Germany; k.moeller@live.de; 3 Digestive Endoscopy Unit, Hopital Privé J Mermoz Ramsay Générale de Santé, 69008 Lyon, France; dr.napoleon@wanadoo.fr; 4 Internal Medicine II, Helios Hospital Meiningen, 98617 Meiningen, Germany; michaelhocke1@aol.com; 5 Medical Department B, University Hospital Münster, 48149 Münster, Germany; barbara.braden@ukmuenster.de; 6 Department for Internal Medicine, Krankenhaus Märkisch Oderland, 15344 Strausberg, Germany; c.jenssen@khmol.de; 7 Brandenburg Institute for Clinical Ultrasound (BICUS), Brandenburg Medical University, 16816 Neuruppin, Germany

**Keywords:** contrast-enhanced ultrasound, endoscopic ultrasound, low mechanical index, CELMI-EUS, focal liver lesions, liver imaging, ultrasound contrast agents, CEUS LI-RADS, hepatocellular carcinoma, liver metastases

## Abstract

Contrast-enhanced low-mechanical-index endoscopic ultrasound (CELMI-EUS) extends the diagnostic capabilities of endoscopic ultrasound by enabling real-time assessment of hepatic microvascularization and perfusion using intravenous ultrasound contrast agents. Owing to its close proximity to the liver and high spatial resolution, CELMI-EUS is particularly suited for the detection and characterization of small or otherwise inconspicuous focal liver lesions. This review summarizes the technical principles of CELMI-EUS, contrast agent usage, vascular phase interpretation, and standardized assessment of benign and malignant focal liver lesions, including the potential and provisional application of CEUS Liver Imaging Reporting and Data System (LI-RADS) terminology in patients at risk for hepatocellular carcinoma, while acknowledging that dedicated validation for CELMI-EUS is lacking. Typical enhancement patterns of common benign lesions such as hemangioma, focal nodular hyperplasia, hepatocellular adenoma, biliary hamartomas, and abscesses are discussed, as well as malignant entities including metastases, hepatocellular carcinoma, and cholangiocarcinoma. In addition, the role of CELMI-EUS in lesion detection, interventional guidance, and its limitations and pitfalls are addressed. Integrated into a multimodality imaging approach alongside transcutaneous CEUS and cross-sectional imaging, CELMI-EUS represents a valuable complementary tool that improves diagnostic confidence and supports optimized management of patients with focal liver lesions.

## 1. Introduction

Contrast-enhanced low-mechanical-index endoscopic ultrasound (CELMI-EUS) enables real-time evaluation of hepatic microvascularization and perfusion by combining high-resolution endoscopic ultrasound with time–intensity curve (TIC) analysis after intravenous administration of ultrasound contrast agents [1]. The use of a low mechanical index preserves microbubble integrity, allowing continuous dynamic assessment of enhancement patterns. In the liver, CELMI-EUS is particularly valuable for the characterization of focal liver lesions, assessment of tumor vascularity, and detection of small lesions not readily accessible or visible with transabdominal imaging [2,3,4,5,6,7,8,9,10,11,12,13,14]. CELMI-EUS is particularly indicated when transcutaneous CEUS or cross-sectional imaging is inconclusive or limited.

## 2. Comparison of Transcutaneous CEUS and CELMI-EUS

Comparison of transcutaneous CEUS and CELMI-EUS highlights important technical differences that influence diagnostic performance. While transcutaneous CEUS is well established for the detection and characterization of focal liver lesions, its sensitivity may be limited by depth, acoustic window, obesity, bowel gas, and respiratory motion. In addition, the use of higher acoustic output and repeated insonation during transcutaneous scanning can lead to microbubble destruction, particularly in deep-seated lesions, thereby reducing contrast persistence and lesion conspicuity. In contrast, CELMI-EUS benefits from close proximity of the transducer to the liver and consistently low-mechanical-index imaging, which preserves microbubble integrity and allows continuous real-time assessment of enhancement patterns. Although endoscopic transducers operate at higher frequencies, the short insonation distance compensates for attenuation and enables superior spatial resolution and improved lesion-to-parenchyma contrast, particularly for small or otherwise inconspicuous lesions. As a result, CELMI-EUS may improve lesion conspicuity and facilitate detection of selected small lesions located close to the transducer; however, comparative evidence remains limited.

Overall, transabdominal CEUS remains the preferred first-line contrast-enhanced ultrasound technique for comprehensive evaluation of focal liver lesions because of its whole-liver coverage and extensive evidence base. CELMI-EUS should be considered a complementary problem-solving modality in selected patients requiring higher spatial resolution, evaluation of indeterminate lesions, or simultaneous tissue acquisition. The following section summarizes the clinical scenarios in which CELMI-EUS provides the greatest additional diagnostic value.

## 3. Examination Technique in CELMI-EUS for Liver Imaging

CELMI-EUS of the liver is performed using a linear-array echoendoscope equipped with contrast-specific imaging modes. After standard patient preparation for EUS, the examination is conducted from the stomach and duodenum, allowing close proximity to the left and right hepatic lobes, respectively. The left liver lobe is typically assessed from the stomach, while the right lobe and hepatic hilum can be evaluated from the duodenal bulb or descending duodenum, depending on individual anatomy [2].

A thorough baseline examination using B-mode and color Doppler imaging is mandatory prior to contrast administration to localize focal liver lesions, assess echogenicity, margins, and vascular relationships, and to optimize probe position. Intravenous administration of an ultrasound contrast agent is then performed as a bolus injection followed by a saline flush. Imaging is carried out using a low mechanical index, which preserves microbubble integrity and enables continuous real-time visualization of contrast enhancement.

Dual-screen display with simultaneous B-mode and contrast imaging is recommended to maintain anatomical orientation and lesion tracking. The liver is observed dynamically throughout the arterial (10–45 s), portal venous (30–120 s), and late phases (>120 s), with particular attention to enhancement pattern, vascular architecture, and washout timing. Care should be taken to minimize excessive probe motion and acoustic output to avoid microbubble destruction [15,16]. Reinjection of contrast may be performed if additional lesions are suspected or if further characterization is required.

CELMI-EUS combines high spatial resolution with stable contrast visualization and is particularly suited for the assessment of small or otherwise inconspicuous liver lesions, as well as for guiding targeted biopsy or interventional procedures when indicated.

### 3.1. Improved Detection of Focal Liver Lesions by CELMI-EUS

Beyond lesion characterization, CELMI-EUS may improve the detection of focal liver lesions by enhancing lesion-to-parenchyma contrast in real time [11]. The continuous low-MI technique preserves microbubble integrity, allowing prolonged observation of arterial, portal venous, and late-phase enhancement, which facilitates the identification of small, deeply located, or otherwise inconspicuous lesions. In particular, malignant liver lesions typically demonstrate contrast washout in the portal venous and late phases, rendering lesions visible that may be missed on conventional B-mode EUS or transabdominal ultrasound. Owing to its close proximity to the liver and high spatial resolution, CELMI-EUS is especially advantageous for detecting small metastases and lesions in difficult anatomical locations [11].

### 3.2. Practical Tips for Beginners Performing CELMI-EUS of Focal Liver Lesions

Although the fundamental principles of contrast-enhanced endoscopic ultrasound are similar to those of transabdominal CEUS, several technical aspects are particularly important when performing CELMI-EUS of the liver. Careful adherence to a standardized examination protocol improves image quality, reduces artifacts, and increases diagnostic confidence.

Before contrast administration, a comprehensive conventional EUS examination should always be performed using B-mode imaging and color Doppler. The lesion should be localized, its relationship to vascular structures documented, and the optimal scanning plane established. Once contrast injection has started, unnecessary repositioning of the echoendoscope should be avoided to permit continuous assessment of enhancement kinetics.

A very low mechanical index should be maintained throughout the examination to preserve microbubble integrity. Continuous prolonged insonation should be minimized, particularly during the portal venous and late phases, because high-frequency endoscopic transducers may accelerate microbubble destruction and produce pseudo-washout. Intermittent imaging during the late phase is often preferable for preserving contrast enhancement.

Dual-display imaging, simultaneously showing B-mode and contrast-specific images, facilitates anatomical orientation and helps maintain visualization of small lesions throughout all vascular phases. Stable positioning of the echoendoscope is particularly important for accurate assessment of arterial enhancement and washout timing and also improves reproducibility when quantitative perfusion analysis is performed.

Interpretation should always include comparison of lesion enhancement with the surrounding liver parenchyma rather than relying on the lesion alone. When enhancement patterns remain equivocal because of motion artifacts, suboptimal visualization, or microbubble depletion, reinjection of the contrast agent is recommended rather than drawing conclusions from inadequate imaging.

Finally, CELMI-EUS should be regarded as a complementary imaging modality within a multimodality diagnostic strategy. Findings should always be interpreted together with the clinical context, conventional EUS, transabdominal CEUS, and cross-sectional imaging whenever available. Machine settings should remain unchanged throughout dynamic contrast imaging whenever possible to ensure reliable comparison between vascular phases.

### 3.3. Contrast Agents and Dosing

For CELMI-EUS of the liver, second-generation ultrasound contrast agents (UCAs) composed of gas-filled microbubbles are used. The most commonly applied agents include SonoVue^®^/Lumason^®^ (microbubbles consisting of a phospholipid shell containing sulfur hexafluoride gas) and, in selected regions, Sonazoid^®^ (perfluorobutane microbubbles). These agents remain predominantly intravascular and thereby enable real-time assessment of hepatic microvascularization and perfusion.

UCAs are administered as a rapid intravenous bolus injection, followed immediately by a saline flush. Typical doses are higher compared to transcutaneous CEUS, usually 4.8 mL for SonoVue^®^/Lumason^®^, depending on patient habitus, equipment sensitivity, and diagnostic task. With Sonazoid^®^, lower bolus doses are commonly used, and delayed post-vascular phase imaging may be performed to exploit Kupffer cell uptake.

CELMI-EUS allows repeated contrast reinjection during the same examination, which is particularly useful for evaluation of multiple lesions, clarification of indeterminate findings, or targeted assessment prior to biopsy or intervention. Strict adherence to low-mechanical-index imaging is essential to minimize microbubble destruction and to ensure prolonged visualization of arterial, portal venous, and late-phase enhancement.

### 3.4. Safety and Contraindications

Ultrasound contrast agents (UCAs) used for CELMI-EUS have an excellent safety profile and are well tolerated, with a very low incidence of adverse reactions. As UCAs are not nephrotoxic and are not excreted by the kidneys, impaired renal function is not a contraindication, and no laboratory testing is required prior to administration. Contraindications are limited and include known hypersensitivity to the contrast agent and severe or unstable cardiopulmonary conditions, in which careful risk–benefit assessment is required before use.

## 4. Vascular Phases and Timing: Interpretation Principles

Following intravenous administration of an ultrasound contrast agent, CELMI-EUS of the liver allows real-time assessment of lesion perfusion across distinct but overlapping vascular phases. The arterial phase, typically beginning approximately 10–20 s after injection and lasting up to 30–45 s, reflects hepatic arterial supply and provides critical information on vascular architecture and arterial enhancement patterns. The portal venous phase, occurring roughly between 30–120 s, corresponds to predominant portal venous perfusion of the liver parenchyma and is particularly important for detecting contrast washout in malignant lesions. The late phase, extending beyond 120 s until microbubble clearance, enables sensitive identification of hypoenhancing lesions against the persistently enhanced liver parenchyma.

Interpretation of enhancement behavior relies not only on the presence or absence of washout but also on its timing and degree. Early washout (<60 s) and/or marked hypoenhancement are highly suggestive of non-hepatocellular malignancy, such as metastases or cholangiocarcinoma, whereas late-onset (≥60 s) and typically mild washout is characteristic of hepatocellular carcinoma. Benign focal liver lesions generally show sustained iso- or hyperenhancement without washout throughout the portal venous and late phases. When using contrast agents with post-vascular properties, such as Sonazoid^®^, additional delayed imaging may further improve lesion detection by demonstrating enhancement defects in malignant lesions.

### 4.1. EUS-Specific Considerations for Vascular Phase Interpretation

In contrast to transabdominal CEUS, interpretation of vascular phases during CELMI-EUS requires awareness of technical factors that may influence microbubble behavior. Although CELMI-EUS is performed using a low mechanical index (MI), the higher operating frequencies of endoscopic ultrasound transducers and prolonged continuous insonation may accelerate microbubble destruction, particularly during the portal venous and late phases. Progressive depletion of contrast microbubbles may result in reduced background parenchymal enhancement and apparent lesion hypoenhancement (pseudo-washout), potentially leading to overestimation of true contrast washout if not recognized.

To preserve microbubble integrity and optimize interpretation, several practical measures are recommended (Table 1). The mechanical index should be kept as low as reasonably achievable while maintaining adequate image quality. Unnecessary continuous insonation should be avoided, particularly during the late phase, by using intermittent imaging when clinically appropriate. Acoustic output should be minimized, and unnecessary changes in focal zone, magnification, or output power should be avoided once contrast enhancement has begun. Stable positioning of the echoendoscope is essential to maintain reproducible visualization of lesion enhancement throughout all vascular phases and to facilitate accurate comparison with the surrounding liver parenchyma. If microbubble depletion compromises interpretation or if additional lesions require evaluation, reinjection of the ultrasound contrast agent is appropriate and may restore optimal diagnostic conditions.

These technical considerations are particularly relevant when assessing washout timing and intensity, as these parameters play a central role in differentiating benign from malignant focal liver lesions and in the provisional application of CEUS LI-RADS criteria. Awareness of pseudo-washout caused by microbubble destruction helps prevent diagnostic misinterpretation and improves the reliability of CELMI-EUS examinations.

The continuous real-time imaging capability of CELMI-EUS enables precise temporal assessment of enhancement dynamics and washout thresholds, thereby improving diagnostic confidence and standardization of lesion characterization.

### 4.2. Quantitative Analysis Techniques

Quantitative analysis techniques may further expand the diagnostic potential of CELMI-EUS. Time–intensity curve (TIC) analysis enables objective assessment of enhancement kinetics by measuring parameters such as peak enhancement, wash-in slope, time to peak, and washout dynamics [15,17,18]. Quantitative perfusion assessment may improve differentiation between benign and malignant focal liver lesions and reduce observer dependency compared with purely visual interpretation [7,19]. In addition, objective measurement of washout timing and intensity may support standardized application of CEUS LI-RADS criteria [20,21,22,23,24]. Stable probe position during CELMI-EUS may facilitate quantitative perfusion analysis compared with transabdominal CEUS by reducing respiratory motion and improving reproducibility of time–intensity curves. Emerging approaches including artificial intelligence, radiomics, and automated perfusion analysis may further enhance lesion characterization and diagnostic reproducibility. However, current experience with quantitative CELMI-EUS remains limited, and reproducibility is influenced by factors such as probe stability, respiratory motion, contrast injection technique, and differences in ultrasound equipment and software. Further prospective studies and standardization efforts are required before quantitative CELMI-EUS can be routinely implemented in clinical practice. Future developments are expected to include fully automated perfusion analysis, radiomics, and artificial intelligence-assisted lesion classification.

## 5. Pitfalls and Limitations

Comprehensive examination of the entire liver using CELMI-EUS is rarely feasible. In particular, lesions located in the posterosuperior segments (segments VII and VIII) may be difficult or impossible to access due to anatomical limitations.

Despite its high spatial resolution and real-time imaging capabilities, CELMI-EUS of the liver has several limitations that must be recognized to avoid diagnostic misinterpretation. Overlap of enhancement patterns may occur between benign and malignant lesions, particularly between hepatocellular adenoma and well-differentiated hepatocellular carcinoma, as both entities can demonstrate arterial hyperenhancement with absent or minimal washout. In such cases, imaging findings should be interpreted in conjunction with clinical context, laboratory data, and, when necessary, histopathology.

Technical artifacts may further complicate interpretation. Pseudo washout can result from microbubble destruction due to excessive acoustic output or prolonged continuous insonation, leading to apparent late-phase hypoenhancement that does not reflect true lesion washout. Motion artifacts, including respiratory movement and endoscope instability, may impair assessment of early arterial enhancement, while blooming artifacts in strongly hyperenhancing lesions can obscure lesion margins and internal architecture.

In addition, CELMI-EUS has an inherently limited field of view compared with transcutaneous CEUS, restricting comprehensive whole-liver screening. Lesions located in peripheral segments or areas inaccessible to the echoendoscope may be missed. Therefore, CELMI-EUS should be regarded as a complementary technique rather than a replacement for transcutaneous ultrasound or cross-sectional imaging. Awareness of these pitfalls and careful optimization of scanning technique are essential for accurate lesion characterization.

## 6. Clinical Scenarios and Appropriate Use of CELMI-EUS

Contrast-enhanced low-mechanical-index endoscopic ultrasound (CELMI-EUS) should be regarded as a complementary imaging modality rather than a replacement for transabdominal CEUS or cross-sectional imaging. Its principal strength lies in the evaluation of selected clinical scenarios in which conventional imaging remains inconclusive or technically limited. Owing to the close proximity of the echoendoscope to the liver, high spatial resolution, and real-time assessment of microvascular perfusion, CELMI-EUS may provide additional diagnostic information that directly influences patient management.

CELMI-EUS is particularly valuable for the evaluation of small lesions adjacent to the stomach or duodenum, lesions detected during staging EUS, patients with poor transabdominal acoustic windows due to obesity or bowel gas, and situations in which lesion characterization and tissue acquisition can be performed during the same procedure. Conversely, transabdominal CEUS remains the preferred first-line technique for comprehensive whole-liver evaluation because of its broader field of view and accessibility.

Table 2 summarizes the principal clinical indications and complementary role of CELMI-EUS in routine practice.

## 7. Characterization of Focal Liver Lesions

### 7.1. Benign Versus Malignant FLL

Characterization of focal liver lesions (FLLs) aims primarily to differentiate benign from malignant entities in order to guide appropriate clinical management. Benign FLLs typically show stable size, well-defined margins, and characteristic enhancement patterns that reflect preserved or organized vascular architecture, whereas malignant lesions are characterized by neoangiogenesis, disorganized microvasculature, and progressive contrast washout [25] The typical enhancement patterns of the most commonly observed focal liver lesions are detailed in Table 3. Contrast-enhanced ultrasound techniques, including CELMI-EUS, enable real-time assessment of vascular behavior across arterial, portal venous, and late phases, which is critical for this distinction. The presence of arterial hyperenhancement followed by portal venous or late-phase washout is highly suggestive of malignancy, while sustained iso- or hyperenhancement relative to surrounding liver parenchyma is more consistent with benign lesions. Accurate differentiation between benign and malignant FLLs reduces unnecessary biopsies and interventions while ensuring timely diagnosis and treatment of malignant disease.

### 7.2. Benign FLL

#### 7.2.1. Hemangioma

Hemangioma is the most common benign focal liver lesion and is typically detected incidentally in asymptomatic patients. On ultrasound, hemangiomas often appear as well-defined, homogeneously hyperechoic lesions, although echogenicity may vary with lesion size and background liver parenchyma.

Contrast-enhanced ultrasound, including CELMI-EUS, provides characteristic vascular patterns that may support a confident diagnosis when the complete typical pattern is present. Typical findings include peripheral, nodular, discontinuous arterial enhancement with progressive centripetal fill-in and persistent iso- or hyperenhancement during the portal venous and late phases. These hallmark enhancement features reflect the cavernous vascular architecture of hemangiomas and help distinguish them from malignant lesions, which demonstrate contrast washout. Accurate recognition of these patterns avoids unnecessary biopsy or further cross-sectional imaging [25].

When assessed with contrast-enhanced low-mechanical-index endoscopic ultrasound (CELMI-EUS), hepatic hemangiomas benefit from the high spatial resolution and close proximity of the endoscopic transducer to the liver. The low mechanical index preserves microbubble integrity, enabling continuous real-time visualization of the typical peripheral nodular arterial enhancement and progressive centripetal fill-in without premature microbubble destruction. This is particularly advantageous for small hemangiomas, atypical lesions, or lesions located in deep or subcapsular regions that may be inconspicuous on transabdominal ultrasound. Persistent iso- or hyperenhancement in the portal venous and late phases on CELMI-EUS supports a benign diagnosis in the appropriate clinical context and helps differentiate hemangiomas from hypervascular malignant lesions, which characteristically exhibit contrast washout.

Specific Advantages of CELMI-EUS: The close proximity of the echoendoscope to the liver enables excellent visualization of the characteristic peripheral nodular enhancement and progressive centripetal fill-in, particularly in small hemangiomas or lesions adjacent to the stomach or duodenum. The higher spatial resolution of CELMI-EUS facilitates delineation of lesion margins and vascular architecture that may be difficult to appreciate with transabdominal CEUS. In selected cases, these features increase diagnostic confidence and help avoid unnecessary biopsy or additional cross-sectional imaging.

#### 7.2.2. Focal Nodular Hyperplasia (FNH)

**Figure 1:**
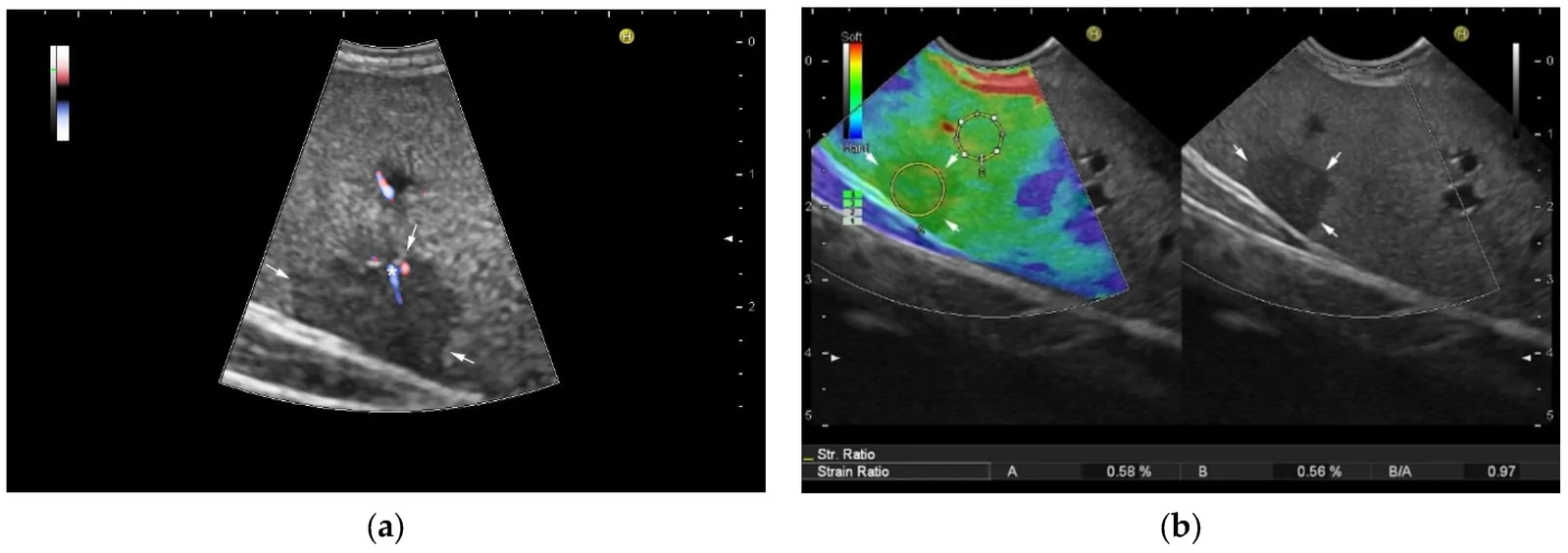

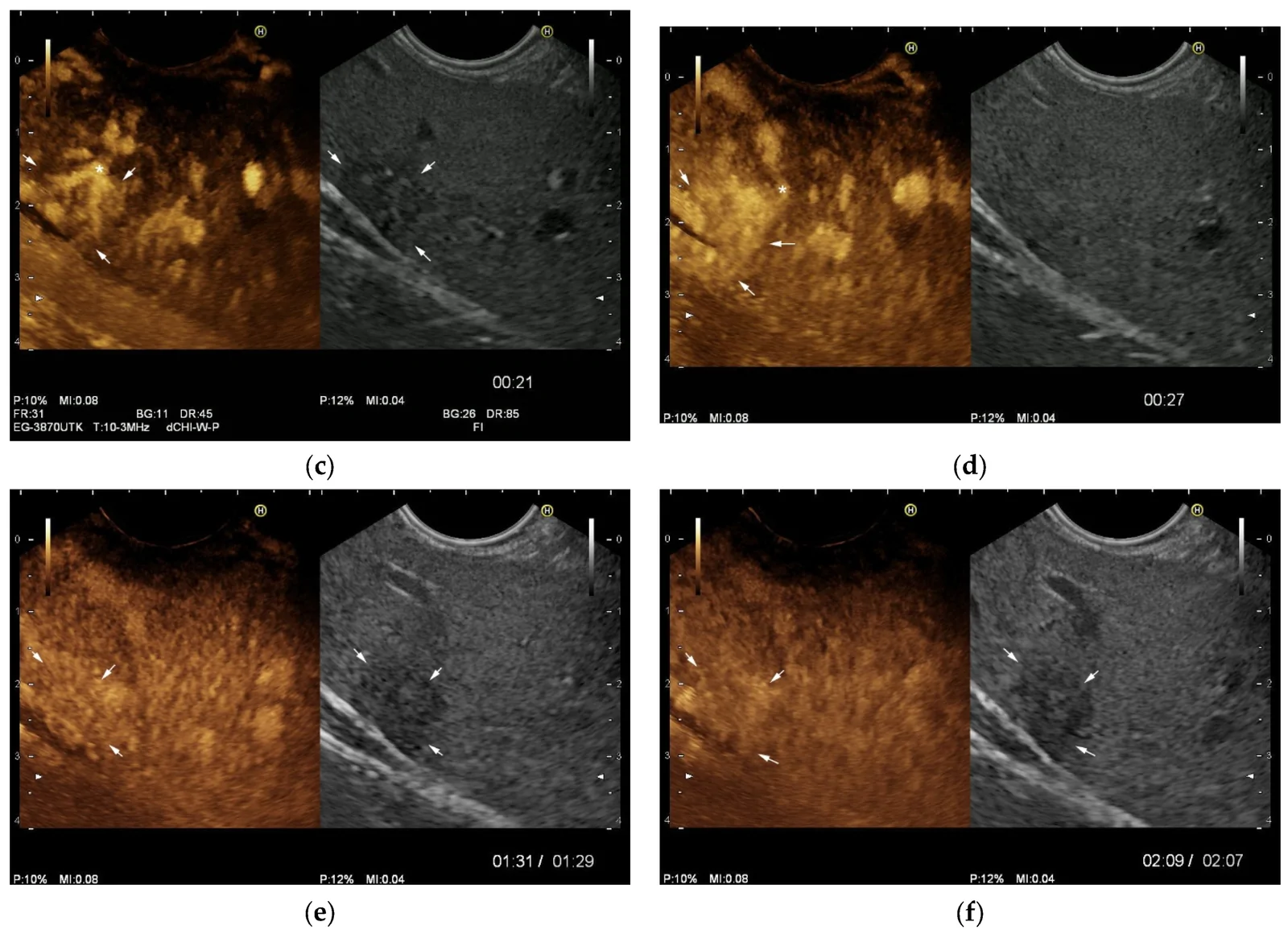
Focal Nodular Hyperplasia. Multiparametric EUS characterisation of a hypoechoic solid FLL with a diameter of 12 × 9 mm found on endoscopic ultrasound in a patient examined for staging of non-small cell lung cancer: B-Mode and Colour Doppler showing a branching vessel entering the lesion ((**a**), feeding and draining vessels marked with centered arrow and *). Strain elastography demonstrating equal stiffness of the lesion compared to surrounding liver tissue with a strain ratio of 0.97 (**b**). CE-EUS demonstrating blood inflow from a peripherally located branching vessel into the lesion in early arterial phase ((**c**); vessel marked with *), diffuse hyperenhancement of the lesion (marked with arrows) only 6 s later in the later arterial phase (**d**) and persisting hyperenhancement also in the late phase 90 and 129 s post injection of the ultrasound contrast agent SonoVue^®^ (Bracco, Konstanz, Germany) (**e**,**f**). The sustained enhancement pattern strongly supported a benign hepatocellular lesion and was considered characteristic of focal nodular hyperplasia in the clinical context.

Focal nodular hyperplasia (FNH) is a benign hyperplastic liver lesion characterized by preserved hepatocellular architecture and a central feeding artery. On contrast-enhanced low-mechanical-index endoscopic ultrasound (CELMI-EUS), FNH typically demonstrates rapid, intense, and homogeneous arterial hyperenhancement, often with a centrifugal “spoke-wheel” vascular pattern originating from a central artery. Owing to the preservation of normal portal venous and sinusoidal perfusion, FNH remains iso- or mildly hyperenhancing during the portal venous and late phases, without contrast washout [Figure 1]. Visualization of the central feeding artery and centrifugal vascular branching may be superior because of the close proximity of the echoendoscope. The continuous real-time imaging enabled by CELMI-EUS allows optimal visualization of early arterial vascular architecture and sustained enhancement, facilitating confident differentiation from hepatocellular adenoma and malignant lesions, which characteristically exhibit washout. This diagnostic confidence may reduce the need for biopsy or further cross-sectional imaging [25].

Specific Advantages of CELMI-EUS: CELMI-EUS is particularly well suited for demonstrating the central feeding artery and centrifugal spoke-wheel vascular architecture because of its superior spatial resolution and near-field imaging capabilities. Continuous real-time assessment of arterial enhancement facilitates differentiation from hepatocellular adenoma and hypervascular malignant lesions. These advantages are most pronounced for small lesions or lesions incompletely characterized by transabdominal imaging.

#### 7.2.3. Hepatocellular Adenoma (HCA)

Hepatocellular adenoma (HCA) is a benign hepatocellular tumor with variable imaging features that reflect its heterogeneous histopathological subtypes. On contrast-enhanced low-mechanical-index endoscopic ultrasound (CELMI-EUS), HCA typically demonstrates rapid and often homogeneous arterial hyperenhancement, frequently with a peripheral-to-central filling pattern and without the spoke-wheel architecture characteristic of focal nodular hyperplasia. During the portal venous and late phases, most HCAs show isoenhancement or mild washout, which may overlap with malignant lesions and limits definitive noninvasive diagnosis in some cases. CELMI-EUS enables real-time assessment of enhancement dynamics and intralesional heterogeneity, including nonenhancing areas related to hemorrhage or necrosis, which can support lesion characterization and guide targeted biopsy when required. CELMI-EUS facilitates visualization of intralesional heterogeneity and viable tissue, which may help guide biopsy when imaging findings remain equivocal. Given the potential risk of hemorrhage and malignant transformation, particularly in larger lesions, imaging findings must be interpreted in clinical context [26].

Specific Advantages of CELMI-EUS: The high spatial resolution of CELMI-EUS improves visualization of intralesional heterogeneity, hemorrhagic components, and viable vascularized tissue. Contrast enhancement may facilitate identification of the most representative areas for EUS-guided tissue acquisition when imaging findings remain indeterminate. Consequently, CELMI-EUS serves as a valuable adjunct in patients in whom differentiation from well-differentiated hepatocellular carcinoma remains uncertain.

#### 7.2.4. Abscess

Liver abscess represents an infectious focal liver lesion with characteristic perfusion patterns on contrast-enhanced imaging. On contrast-enhanced low-mechanical-index endoscopic ultrasound (CELMI-EUS), abscesses typically demonstrate peripheral rim-like arterial enhancement with complete nonenhancement of the central liquefied or necrotic cavity. Enhancing septations may be present, while the lesion commonly becomes hypoenhancing in the portal venous and late phases. In contrast to necrotic malignant tumors, which usually show irregular intralesional enhancement of viable tumor tissue followed by contrast washout, abscesses lack internal vascularized solid components and display smooth or lobulated inflammatory rims. Recognition of these features on CELMI-EUS enables reliable differentiation from necrotic metastases or hepatocellular carcinoma and supports appropriate clinical management.

#### 7.2.5. Von Meyenburg Complexes

Von Meyenburg complexes (biliary hamartomas) are benign developmental malformations of the intrahepatic bile ducts, typically presenting as multiple small, irregularly distributed lesions and often detected incidentally. On contrast-enhanced low-mechanical-index endoscopic ultrasound (CELMI-EUS), these lesions characteristically show no contrast enhancement in any vascular phase, reflecting their cystic, nonvascular nature. They remain nonenhancing during the arterial, portal venous, and late phases, without washout or progressive filling. CELMI-EUS is particularly useful in confirming the avascular behavior of these lesions and in differentiating them from small metastases or microabscesses, which demonstrate contrast washout. Recognition of their typical nonenhancing pattern avoids misdiagnosis and unnecessary further investigations.

#### 7.2.6. Parasites

Parasitic liver diseases, particularly echinococcosis, may present as focal liver lesions and can mimic benign or malignant tumors on conventional imaging [27,28,29]. Hepatic echinococcosis is caused by infection with *Echinococcus granulosus* (cystic echinococcosis) or, less commonly, *Echinococcus multilocularis* (alveolar echinococcosis). On B-mode ultrasound, cystic echinococcosis typically appears as a complex cystic lesion with internal septations, daughter cysts, detached membranes, or calcifications, whereas alveolar echinococcosis often demonstrates an infiltrative heterogeneous mass-like appearance with irregular margins and mixed solid-cystic components. On contrast-enhanced low-mechanical-index endoscopic ultrasound (CELMI-EUS), echinococcal lesions characteristically show absent intralesional enhancement due to the avascular parasitic cyst content, while peripheral inflammatory or fibrotic tissue may demonstrate rim-like enhancement. In alveolar echinococcosis, irregular peripheral enhancement with central nonenhancing necrotic areas may resemble cholangiocarcinoma or metastases. Recognition of the typical lack of internal vascularization and correlation with clinical, serological, and epidemiological findings are essential for accurate diagnosis and avoidance of unnecessary biopsy or intervention.

#### 7.2.7. Other Benign FLL

Other benign focal liver lesions (FLLs) encompass a heterogeneous group of entities, including focal fatty infiltration or sparing, inflammatory pseudotumors, regenerative nodules in non-cirrhotic liver, simple cysts, and infectious or post-traumatic lesions. On contrast-enhanced low-mechanical-index endoscopic ultrasound (CELMI-EUS), these lesions typically demonstrate either no enhancement or enhancement patterns identical to the surrounding liver parenchyma, without contrast washout. Focal fatty changes show persistent isoenhancement throughout all vascular phases, while simple cysts and necrotic components remain completely non-enhancing. CELMI-EUS allows real-time confirmation of benign vascular behavior and helps exclude malignancy, particularly in lesions with atypical B-mode appearance or in patients with known extrahepatic malignancy. The close proximity of the echoendoscope to the liver, combined with high spatial resolution and continuous low-mechanical-index imaging, enables confident evaluation of small or otherwise inconspicuous lesions and may reduce unnecessary cross-sectional imaging or biopsy when characteristic benign enhancement patterns are demonstrated.

### 7.3. Malignant FLL

#### 7.3.1. Metastases

**Figure 2:**
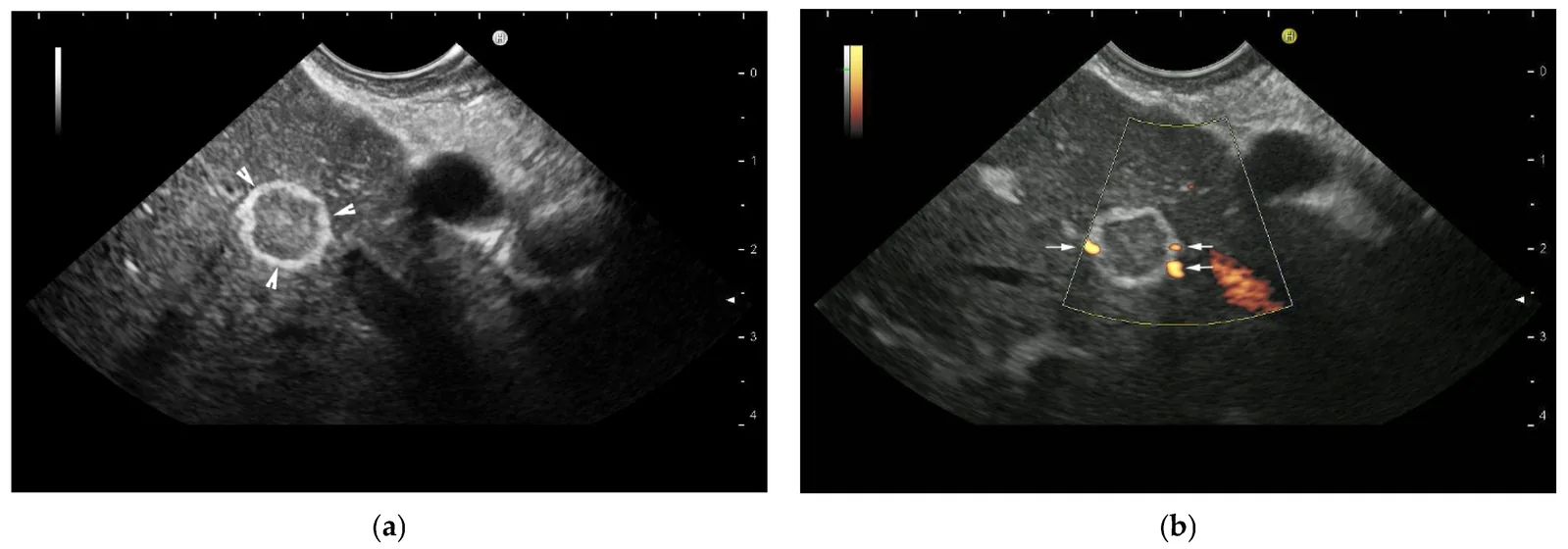

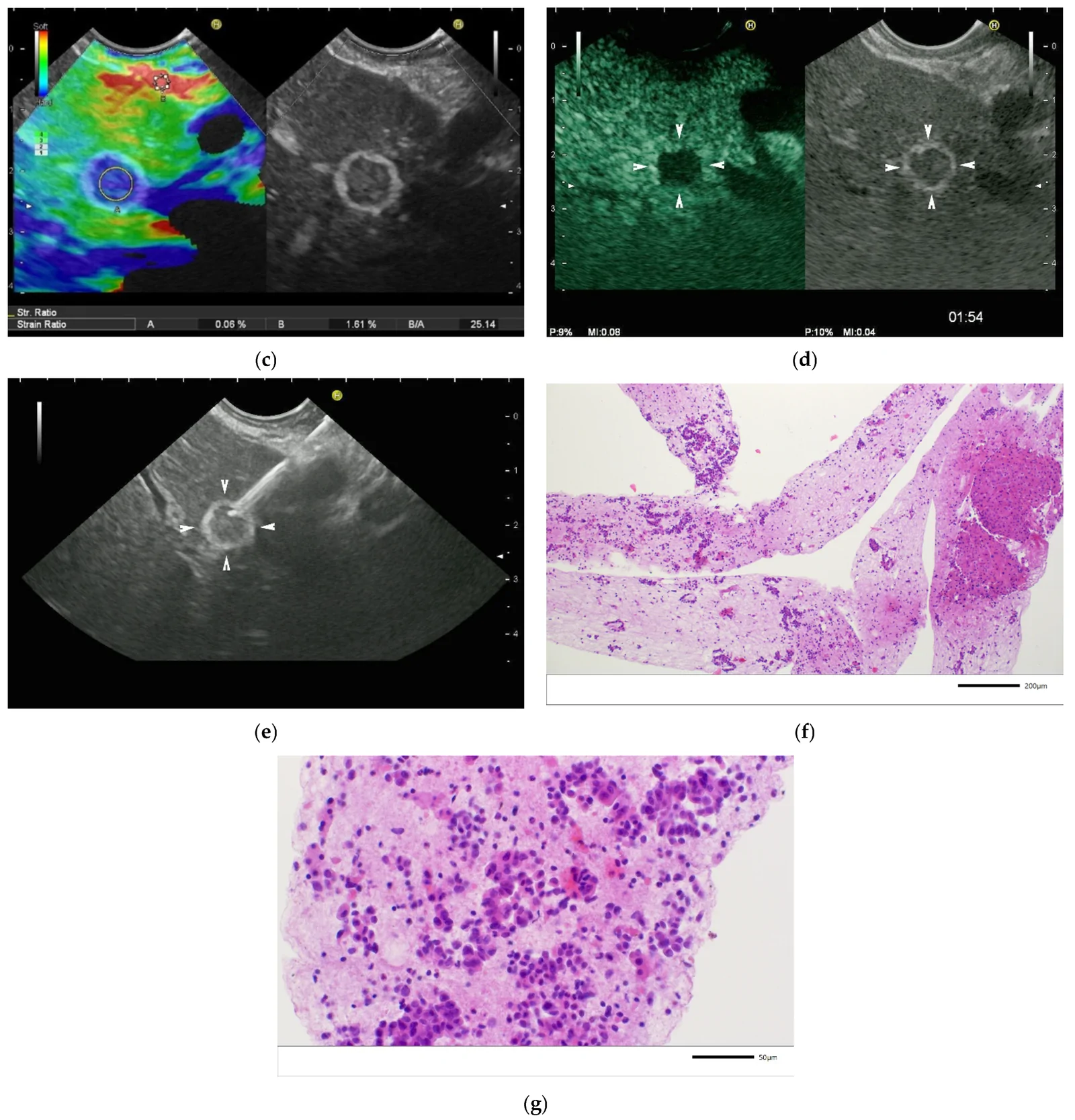
Metastasis. Multiparametric EUS characterisation of a focal liver lesion in a female patient with peripheral pulmonary nodules and a history of breast cancer. A small isoechoic round FLL (11 mm; marked with arrowheads) with a hyperechoic halo was found on endoscopic ultrasound (**a**). Power Doppler revealed a few blood flow signals (arrows) within the halo (**b**). The FLL proved to be very stiff with strain elastography; strain ratio of 25.14 (**c**) and hypoenhancing in the late phase of contrast-enhanced endoscopic ultrasound (marked by arrowheads) (**d**); 1.5 min after injection of the ultrasound contrast agent SonoVue^®^ (Bracco, Konstanz, Germany). This is proof of a malignant FLL, and EUS-guided tissue sampling was performed using a 22G-FNB-needle with a modified Franseen tip design (MICRO-TECH Europe, Düsseldorf, Germany) ((**e**); the tip of the EUS-FNB needle is clearly visible inside the small FLL). The specimen contains large intact cores of liver and tumour tissue ((**f**,**g**); haematoxylin-eosin staining; histopathological images by courtesy of Gunnar Schröder, Institute for Pathology, Königs Wusterhausen, Germany). Immunohistochemical staining revealed a TTF-1 positive adenocarcinoma with no expression of GATA or Estrogen receptor. Finally, the diagnosis of a second cancer of the lung with liver metastasis was made.

Liver metastases represent the most common malignant focal liver lesions and arise from a wide range of primary tumors, most frequently of gastrointestinal, pancreatic, breast, or pulmonary origin [25]. On contrast-enhanced low-mechanical-index endoscopic ultrasound (CELMI-EUS), metastases characteristically demonstrate early and marked contrast washout during the portal venous and late phases, rendering them conspicuous against the enhancing liver parenchyma. Arterial phase enhancement is variable and may be peripheral, rim-like, heterogeneous, or occasionally hypervascular, depending on the primary tumor. The continuous real-time imaging provided by CELMI-EUS enables sensitive detection of small or otherwise inconspicuous metastatic lesions, particularly in deep segments or near the gastrointestinal tract. The presence of rapid and pronounced washout is a key diagnostic feature that reliably differentiates metastases from benign focal liver lesions [Figure 2] [11].

Specific Advantages of CELMI-EUS: CELMI-EUS offers particular advantages for the detection and characterization of small metastatic deposits located in the left hepatic lobe or adjacent to the stomach and duodenum. The combination of high spatial resolution and real-time contrast imaging improves visualization of subtle intratumoral vascular architecture and early washout, which may increase lesion conspicuity compared with conventional B-mode EUS. Furthermore, CELMI-EUS enables immediate EUS-guided biopsy of viable enhancing tumor tissue during the same procedure, thereby combining diagnosis and tissue acquisition in a single session.

#### 7.3.2. Hepatocellular Carcinoma (HCC)

**Figure 3:**
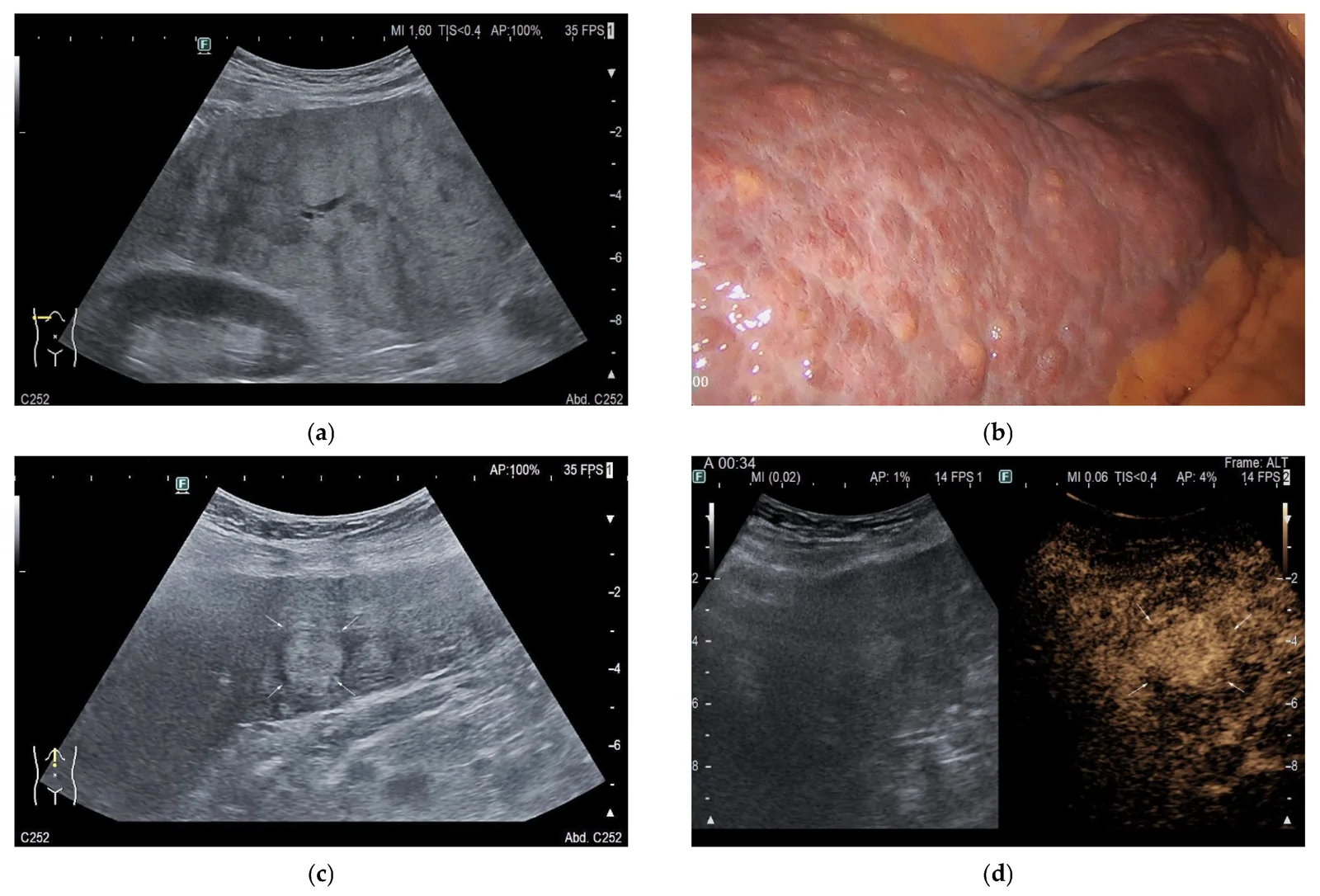

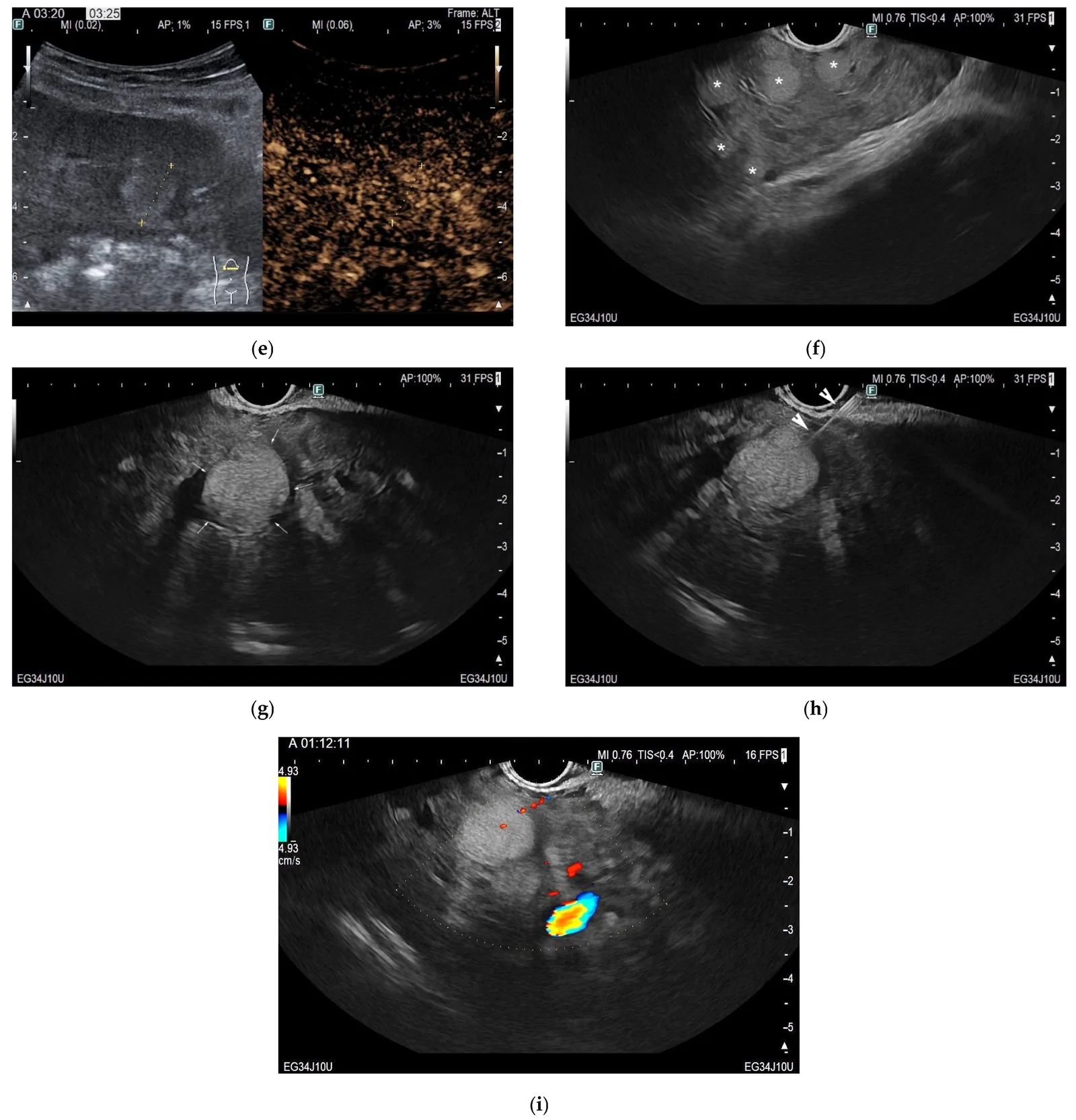
HCC. Patient with liver cirrhosis and right upper quadrant pain, which turned out to be caused by gallbladder stones. The liver showed marked nodularity with multiple, predominantly subcentimeter-sized, round and slightly hyperechogenic focal lesions (**a**), which were later also visualized laparoscopically during cholecystectomy (**b**). The largest echogenic lesion with a diameter of 17 × 20 mm was located in the left hepatic lobe ((**c**); marked with arrows). On magnetic resonance imaging with the liver-specific contrast agent Primovist^®^, all focal lesions were characterized as regenerative nodules. On contrast-enhanced ultrasound, the subcentimeter lesions were isoenhancing in all phases; only the largest lesion was markedly hyperenhanced in the arterial phase ((**d**); marked with arrows; 22 s after injection of the ultrasound contrast agent SonoVue^®^). The hyperenhancement persisted into the late phase ((**e**); lesion between markers; 3 min 25 s after injection), corresponding to a CEUS LI-RADS category LR4. Due to significantly impaired coagulation, the patient’s request for sedation, and the location adjacent to the gastric antrum, EUS-guided sampling was regarded as the most appropriate approach for tissue acquisition. Similar to abdominal ultrasound, endoscopic ultrasound again revealed numerous subcentimeter-sized echogenic nodules ((**f**), marked with *), and the previously described 2 cm echogenic focal lesion could be unequivocally identified ((**g**); marked with arrows). EUS-FNB ((**h**); the 22 G MicroTech Franseen-type needle is marked with arrowheads) yielded adequate material from two needle passes and confirmed the histological diagnosis of a well-differentiated hepatocellular carcinoma. A subclinical hemorrhage visible only on FKDS (“open channel”) ceased after two minutes (*i*).

**Figure 4:**
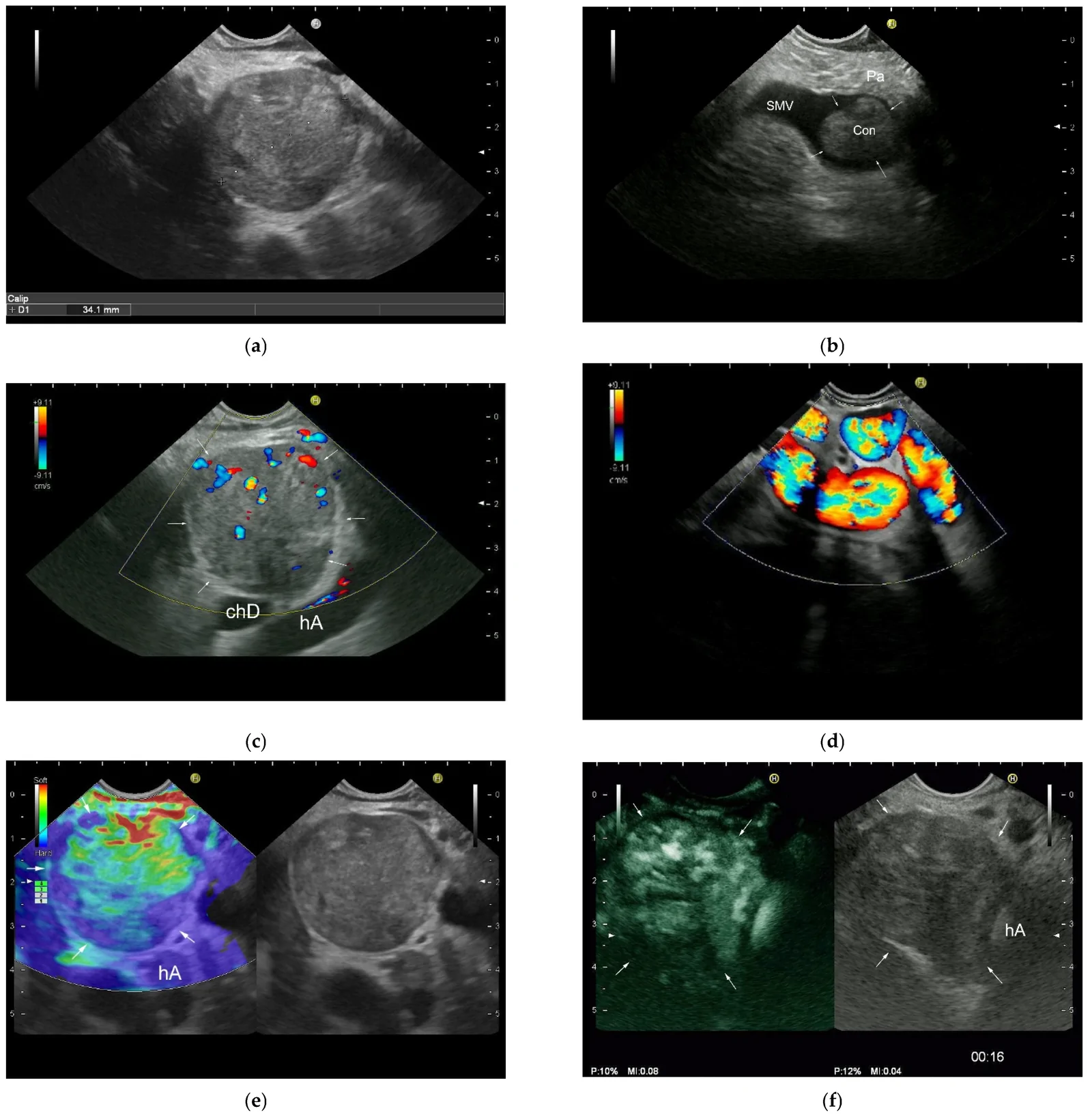

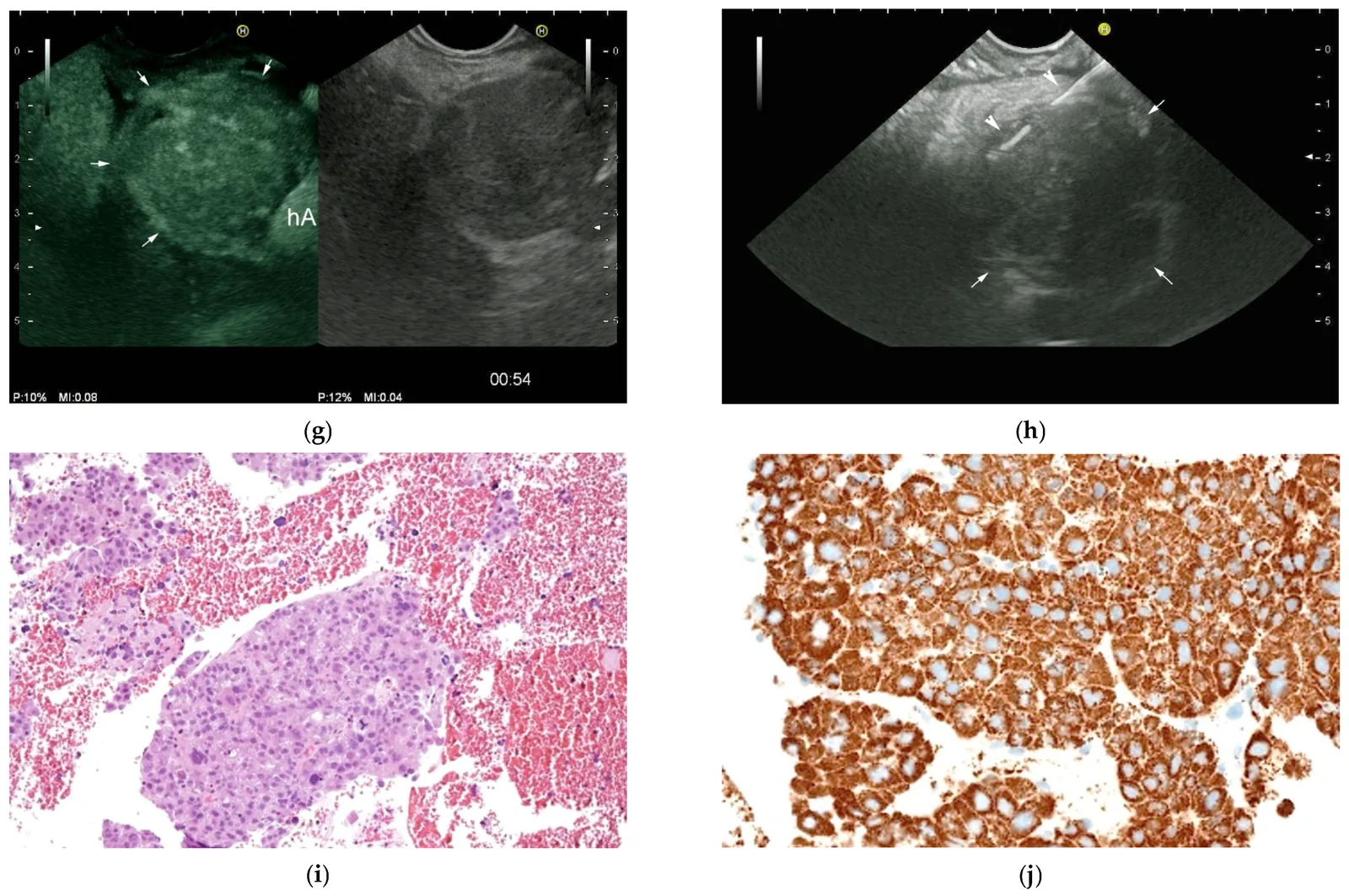
Multiparametric EUS characterization of portal vein tumor thrombus caused by hepatocellular carcinoma. A patient presenting with weight loss underwent transabdominal ultrasound, which demonstrated liver cirrhosis and portal vein thrombosis without an identifiable focal liver lesion. Endoscopic ultrasound revealed marked dilatation of the portal vein (34 mm) at the hepatic hilum, completely occupied by a slightly heterogeneous thrombus ((**a**), between markers), extending to the porto-mesenteric confluence (**b**). Color Doppler imaging demonstrated intrathrombotic vascular signals (**c**) and porto-systemic collateral vessels along the gastric wall (**d**). Strain elastography showed heterogeneous stiffness of the thrombus (**e**). Following intravenous administration of SonoVue^®^ (Bracco, Konstanz, Germany), contrast-enhanced low-mechanical-index EUS demonstrated arterial hyperenhancement of the thrombus with irregular intrathrombotic arterial vessels ((**f**), 16 s after injection), confirming neovascularization consistent with malignant portal vein invasion. At the end of the portal venous phase ((**g**), 54 s), the thrombus remained approximately isoenhancing relative to the surrounding liver parenchyma. During prolonged imaging, progressive depletion of microbubbles caused by continuous insonation with the high-frequency EUS transducer reduced background parenchymal enhancement, illustrating the phenomenon of pseudo-washout. Consequently, the characteristic mild and late washout typically observed in hepatocellular carcinoma may become difficult to appreciate or may even be missed if microbubble destruction is not recognized. This case illustrates how progressive microbubble depletion during prolonged high-frequency insonation may obscure true late mild washout and emphasizes the importance of a very low mechanical index, intermittent late-phase imaging, and reinjection when necessary. EUS-guided fine-needle aspiration was subsequently performed using a 22-gauge FNA needle (Medi-Globe, Rohrdorf-Achenmühle, Germany) ((**h**), arrowheads). Histopathological examination ((**i**); hematoxylin-eosin staining) together with strong cytoplasmic HepPar1 immunoreactivity (**j**) confirmed a portal vein tumor thrombus originating from hepatocellular carcinoma (histopathological images courtesy of S. Bartho, Institute of Pathology, Königs Wusterhausen, Germany). Despite extensive imaging, no primary hepatic mass was identified on subsequent transabdominal ultrasound or magnetic resonance imaging. In subfigures (**b**,**c**,**e**–**h**) the outline of the thrombus is marked with arrows. Abbreviations: chD, common hepatic duct; Con, portal vein confluence with the superior mesenteric and splenic veins; hA, hepatic artery; Pa, pancreatic body; SMV, superior mesenteric vein.

Hepatocellular carcinoma (HCC) is the most common primary malignant liver tumor and typically arises in patients with chronic liver disease or cirrhosis. On contrast-enhanced low-mechanical-index endoscopic ultrasound (CELMI-EUS), HCC characteristically demonstrates non-rim arterial phase hyperenhancement (APHE) followed by late-onset and usually mild contrast washout. These enhancement features form the basis of the CEUS Liver Imaging Reporting and Data System (LI-RADS), which standardizes interpretation and reporting. Lesions showing non-rim APHE with late (≥60 s) and mild washout are classified as CEUS LI-RADS 5 (LR-5), indicating definite HCC, whereas early or marked washout suggests non-hepatocellular malignancy (LR-M). Continuous real-time imaging facilitates precise determination of washout onset, which is particularly important when applying CEUS LI-RADS criteria. CELMI-EUS allows continuous real-time assessment of arterial vascular architecture and precise timing of washout, improving diagnostic confidence, particularly for small lesions or lesions inconspicuous on transabdominal ultrasound [Figure 3]. While CEUS LI-RADS enables confident noninvasive diagnosis of HCC, cross-sectional imaging remains required for staging and complications including portal vein thrombosis [Figure 4]. Although CEUS LI-RADS was developed for transabdominal CEUS, its vascular criteria may also be applied to CELMI-EUS, although dedicated validation studies are currently lacking.

Specific Advantages of CELMI-EUS: CELMI-EUS enables high-resolution assessment of arterial hypervascularity and precise temporal evaluation of washout because of stable near-field imaging and continuous real-time observation. It is particularly useful for small lesions that remain inconspicuous on transabdominal ultrasound or are located adjacent to the upper gastrointestinal tract. In addition, CELMI-EUS facilitates targeted biopsy of viable tumor tissue when histological confirmation is required despite characteristic imaging findings.

#### 7.3.3. Intrahepatic Cholangiocarcinoma (ICC)

Intrahepatic cholangiocarcinoma (ICC) is a primary malignant liver tumor arising from the biliary epithelium and often occurring in non-cirrhotic liver. On CELMI-EUS, ICC typically demonstrates peripheral or heterogeneous arterial hyperenhancement, frequently with a rim-like pattern, followed by early (<60 s) and marked contrast washout in the portal venous and late phases. This enhancement behavior reflects the fibrotic stroma and poor portal venous supply of ICC. According to CEUS LI-RADS, lesions with rim arterial enhancement and/or early and pronounced washout are categorized as LR-M, indicating non-hepatocellular malignancy. CELMI-EUS, with its high spatial resolution and continuous real-time imaging, facilitates accurate assessment of arterial enhancement patterns and washout timing, supporting differentiation of ICC from HCC, which typically shows later and milder washout [30,31,32].

Specific Advantages of CELMI-EUS: CELMI-EUS allows detailed visualization of irregular arterial vascular architecture, peripheral rim enhancement, and early marked washout, all of which support differentiation from hepatocellular carcinoma. In patients with indeterminate imaging findings, contrast enhancement also improves targeting of viable tumor tissue for EUS-guided biopsy, particularly in lesions that are difficult to access percutaneously.

## 8. Role of CELMI-EUS in Interventional Procedures

CELMI-EUS plays an important adjunctive role in EUS-guided interventional procedures involving the liver [7,12,13,14]. By providing real-time visualization of tissue perfusion, CELMI-EUS enables precise targeting of viable, vascularized tumor tissue while avoiding necrotic or nonenhancing areas, thereby improving the diagnostic yield of EUS-guided biopsy. This is particularly valuable in heterogeneous lesions, previously nondiagnostic biopsies, or lesions poorly delineated on B-mode imaging alone.

In addition, CELMI-EUS may support therapy planning and assessment, such as delineation of tumor margins prior to local ablative treatments and early evaluation of treatment response by identifying residual enhancing tissue. During follow-up, absence of contrast enhancement within a treated lesion suggests complete necrosis, whereas persistent or recurrent enhancement indicates residual or recurrent viable tumor. Owing to its real-time capability and close proximity to the liver, CELMI-EUS complements transcutaneous CEUS and cross-sectional imaging in selected interventional and follow-up scenarios [22,24].

## 9. Structured Reporting and Standardization

To ensure consistency, reproducibility, and clear communication of findings, structured reporting is recommended for CELMI-EUS of the liver. Reports should systematically document lesion location, size, number, baseline B-mode appearance, arterial enhancement pattern, presence and timing of washout, and final assessment of benignity or malignancy. In patients at risk for hepatocellular carcinoma, CEUS LI-RADS terminology may provide a useful provisional descriptive framework; however, the algorithm was developed and validated for transabdominal CEUS and has not yet been validated for CELMI-EUS [21,33,34,35].

Findings from CELMI-EUS should be integrated with transcutaneous CEUS, CT, and MRI within a multimodality imaging approach. CELMI-EUS serves as a complementary problem-solving tool, particularly for lesions that are indeterminate, small, or poorly visualized on other modalities. While CELMI-EUS may support a confident diagnosis in selected cases, especially for benign lesions or CEUS LI-RADS LR-5 observations, cross-sectional imaging remains essential for comprehensive staging, treatment planning, and longitudinal follow-up. Consistent integration of CELMI-EUS results into multidisciplinary decision-making enhances diagnostic accuracy and optimizes patient management.

**Key Messages**

- CELMI-EUS complements rather than replaces transabdominal CEUS.
- Highest diagnostic value for small lesions adjacent to the gastrointestinal tract.
- Real-time vascular assessment improves lesion characterization.
- Early washout strongly suggests non-hepatocellular malignancy.
- Late mild washout supports HCC but requires clinical correlation.
- Quantitative analysis and AI represent important future developments.
- Dedicated prospective validation studies remain necessary.

**Figure 5:**
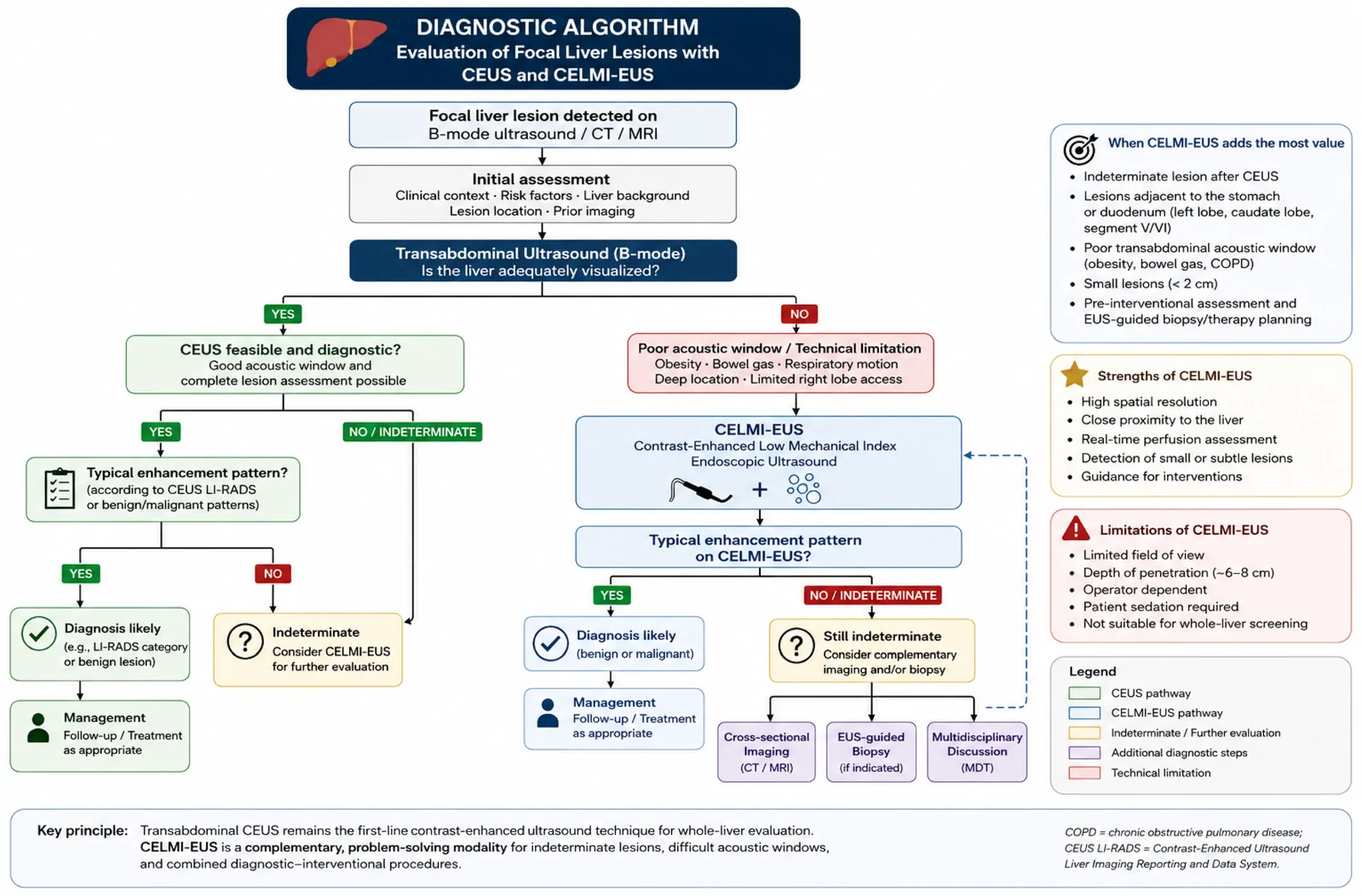
Diagnostic algorithm, flowchart.

Figure 5 summarizes a practical diagnostic algorithm illustrating the complementary roles of transabdominal CEUS, CELMI-EUS, cross-sectional imaging, and biopsy in the evaluation of focal liver lesions.

## 10. Conclusions

CELMI-EUS is a valuable complementary imaging modality for the detection, characterization, and targeted assessment of focal liver lesions. By combining high spatial resolution with continuous real-time evaluation of contrast enhancement, CELMI-EUS enables precise analysis of vascular architecture and washout dynamics, particularly in small, deep, or otherwise inconspicuous lesions. Its ability to differentiate benign from malignant focal liver lesions, apply standardized interpretation frameworks such as CEUS LI-RADS, and support image-guided interventions underscores its clinical relevance.

CELMI-EUS should be integrated into a multimodality imaging strategy, complementing transcutaneous CEUS and cross-sectional imaging rather than replacing them. When applied by experienced examiners and interpreted within the appropriate clinical context, CELMI-EUS improves diagnostic confidence, guides interventional decision-making, and contributes meaningfully to optimized patient care in hepatobiliary imaging.

CELMI-EUS should not be regarded as a replacement for transabdominal CEUS but rather as a complementary high-resolution imaging modality. Its principal value lies in the evaluation of selected focal liver lesions that remain indeterminate after conventional imaging or are optimally accessible from the gastrointestinal tract. Future multicenter prospective studies, standardized quantitative analysis, and dedicated CELMI-EUS validation studies are required before widespread implementation of CELMI-EUS-specific diagnostic algorithms and LI-RADS criteria.

## Figures and Tables

**Table 1:** Practical Recommendations for Vascular Phase Interpretation during CELMI-EUS.

Recommendation	Rationale
Maintain a very low mechanical index	Preserves microbubble integrity
Avoid prolonged continuous insonation	Reduces premature microbubble destruction
Use intermittent imaging during the late phase	Minimizes pseudo-washout
Minimize unnecessary acoustic output adjustments	Maintains stable contrast behavior
Stabilize the echoendoscope	Improves reproducibility and washout assessment
Repeat contrast injection if necessary	Restores optimal enhancement for lesion characterization

**Table 2:** Clinical Scenarios and Appropriate Use of CELMI-EUS.

Clinical Scenario	Added Value of CELMI-EUS	Preferred Imaging Approach
Indeterminate focal liver lesion after transabdominal CEUS and MRI, difficult to access for percutaneous ultrasound-guided sampling	High-resolution assessment of vascular architecture and enhancement kinetics; may improve lesion characterization.	CELMI-EUS
Poor transabdominal acoustic window (obesity, bowel gas, postoperative anatomy)	Close proximity to the liver minimizes attenuation and improves visualization.	CELMI-EUS
Small lesions (<2 cm), particularly in the left lobe or adjacent to the stomach/duodenum	Superior spatial resolution and lesion conspicuity facilitate detection and characterization.	CELMI-EUS
Lesions adjacent to the gastrointestinal tract	Near-field imaging enables excellent visualization of vascular morphology and lesion margins.	CELMI-EUS
Need for EUS-guided tissue acquisition or intervention	Contrast enhancement assists target selection, identification of viable tumor tissue, and procedural planning.	CELMI-EUS
Comprehensive whole-liver screening	Preferred first-line technique because of its larger field of view and complete liver coverage.	Transabdominal CEUS
Typical enhancement pattern confidently established on transabdominal CEUS	Additional CELMI-EUS is usually unnecessary unless biopsy or intervention is planned.	Transabdominal CEUS
Persistent diagnostic uncertainty after ultrasound examinations	Multimodality imaging and, when appropriate, image-guided biopsy provide definitive diagnosis.	CT/MRI ± CELMI-EUS

Key message: Transabdominal CEUS remains the first-line contrast-enhanced ultrasound technique for most focal liver lesions. CELMI-EUS is a complementary problem-solving modality that is particularly valuable for indeterminate lesions, difficult acoustic windows, small lesions adjacent to the gastrointestinal tract, and combined diagnostic-interventional procedures.

**Table 3:** Typical CELMI-EUS Enhancement Patterns of Common Focal Liver Lesions.

Lesion	Arterial	Portal	Late	Key Discriminator	Main CELMI-EUS Advantage
Hemangioma	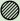 Peripheral nodular	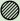 Fill-in	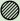 No washout	Persistent enhancement	Excellent depiction of nodules/fill-in
FNH	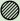 Homogeneous + spoke-wheel	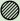 Iso	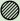 No washout	Spoke-wheel artery	Central artery visualization
HCA	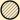 Hyperenhancement	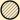 Usually iso	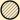 Mild/variable washout	Intralesional heterogeneity	Targeted biopsy guidance
Abscess	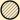 Rim enhancement	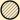 Mild rim washout	◯ Non-enhancing cavity	Avascular necrotic center	Differentiates from necrotic tumors
Von Meyenburg complex	◯ None	◯ None	◯ None	Completely avascular	Avoids confusion with metastases
Parasitic lesions	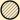 Peripheral rim	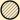 Minimal	◯ Avascular center	No internal vascularity	Defines inflammatory margins
Other benign FLL	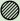 Iso	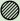 Iso	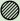 No washout	Benign vascular behavior	May avoid biopsy
Metastases	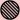 Variable/rim	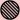 Early washout	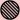 Marked hypo	Early marked washout	Sensitive small-lesion detection
HCC	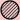 Non-rim APHE	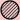 Mild washout ≥60 s	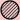 Mild late washout	Late mild washout	Supports CEUS LI-RADS
CCC	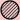 Rim/heterogeneous	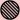 Early washout	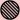 Marked hypo	Early marked washout	Differentiates from HCC

Legend: Green = benign enhancement pattern; Red = malignant enhancement pattern; Yellow = variable/intermediate findings. The key discriminator column summarizes the single most useful diagnostic feature for routine interpretation. Abbreviations: APHE, arterial phase hyperenhancement; CCC, Cholangiocarcinoma; CELMI-EUS, contrast enhanced low mechanical index-endoscopic ultrasound; CEUS, contrast-enhanced ultrasound; FLL; focal liver lesion; FNH, focal nodular hyperplasia; HCA, hepatocellular adenoma; HCC, hepatocellular carcinoma; LI-RADS, Liver Imaging Reporting and Data System.

## Data Availability

No new data were created or analyzed in this study. Data sharing is not applicable to this article.
